# Application of Innovative TGA/Chemometric Approach for Forensic Purposes: The Estimation of the Time since Death in Contaminated Specimens

**DOI:** 10.3390/diagnostics11010121

**Published:** 2021-01-14

**Authors:** Roberta Risoluti, Giuseppina Gullifa, Vittorio Fineschi, Paola Frati, Stefano Materazzi

**Affiliations:** 1 Department of Chemistry, “Sapienza” University of Rome, p.le A.Moro 5, 00185 Rome, Italy; roberta.risoluti@uniroma1.it (R.R.); giuseppina.gullifa@uniroma1.it (G.G.); 2 Department of Anatomical, Histological, Forensic Medicine and Orthopaedic Sciences, “Sapienza” University of Rome, p.le A.Moro 5, 00185 Rome, Italy; vittorio.fineschi@uniroma1.it (V.F.); paola.frati@uniroma1.it (P.F.)

**Keywords:** vitreous humor, postmortem interval, thermogravimetry, chemometrics, forensic pathology

## Abstract

Chronothanatology has always been a challenge in forensic sciences. Therefore, the importance of a multidisciplinary approach for the characterization of matrices (organs, tissues, or fluids) that respond linearly to the postmortem interval (PMI) is emerging increasingly. The vitreous humor is particularly suitable for studies aimed at assessing time-related modifications because it is topographically isolated and well-protected. In this work, a novel approach based on thermogravimetry and chemometrics was used to estimate the time since death in the vitreous humor and to collect a databank of samples derived from postmortem examinations after medico–legal evaluation. In this study, contaminated and uncontaminated specimens with tissue fragments were included in order to develop a classification model to predict time of death based on partial least squares discriminant analysis (PLS-DA) that was as robust as possible. Results demonstrate the possibility to correctly predict the PMI even in contaminated samples, with an accuracy not lower than 70%. In addition, the correlation coefficient of the measured versus predicted outcomes was found to be 0.9978, confirming the ability of the model to extend its feasibility even to such situations involving contaminated vitreous humor.

## 1. Introduction

Recently, the ophthalmologic literature has highlighted the important role of the vitreous humor, which is not simply “the gelatinous fluid situated in the back of the eye” but rather is characterized by a complex composition of analytes and metabolites that are site-specific and that change in relation to the different functions in which they are locally involved [1,2,3]. It is possible to extend the applicability of the vitreous studies to a wide range of time from death (up to 144 h) since chemical modifications occur very slowly [4,5,6,7].

To date, all the analytes studied and recovered from the vitreous humor have shown a concentration gradient controlled by the blood–retinal barrier (BRB) and influenced by the activity of numerous transporters that are Na+/K+ ATP-dependent [8]. Among these, potassium is the major intracellular electrolyte, exhibiting a constant concentration maintained by an active mechanism of Na/K ATPase. In addition, upon death, this pump stops working and potassium reaches extracellular fluid through the cell walls, where its concentration increases. The vitreous is, therefore, valuable for postmortem determination of potassium concentration, being more easily obtainable from the corpse compared to other organs [9] or fluids, such as the cerebrospinal fluid [10,11] and also because it is less perishable than the blood, which quickly undergoes hemolysis. The search for potassium levels in the vitreous has been reported since 1960, with subsequent changes in the models of the spread from the cells to the vitreous. Many authors have extended the investigation to the dosage of carbohydrates, nitrogen compounds, enzymes, proteins, and ions in order to identify a significant relationship between the changes in their concentration and the time elapsed since death [12]. 

Recently, the thermogravimetry associated with chemometric analysis has been proposed as a novel technique that is able to analyze complex matrices [13,14,15,16] because it permits qualitative and quantitative analysis [17,18,19,20,21,22] without any pretreatment. In addition, this approach provided promising results for the estimation of a PMI in the vitreous humor [23,24]. 

In this work, the application of thermogravimetry coupled to chemometrics was investigated to develop a novel classification model to estimate the postmortem interval (PMI) in the vitreous humor by collecting a database of samples and including even contaminated samples with tissue fragments and blood. As the possibility of estimating the PMI in vitreous specimens is often related to their integrity or contaminations, this study represents an update of the methodologies for investigating the time since death, extending the time of prediction to 15 days by using a model able to be used in all such situations involving contaminated vitreous humor from cadavers. 

## 2. Materials and Methods

### 2.1. Humor Specimens

Vitreous specimens were collected during medico–legal autopsies through a scleral puncture on the lateral canthus of each eye (about 2 mL). All the samples from right and left eyes were immediately stored at −20 °C prior to the thermogravimetric analysis in order to avoid samples as a function of the time elapsed between sampling and analysis.

In this work, all the collected samples were processed—even contaminated specimens previously discarded [23,24] due to the interference of the tissue fragments. In particular, a total of 202 postmortem bodies were investigated, resulting in 404 humor samples from both males and females (mean age 68 ± 19 years) during forensic autopsies at the Institute of Forensic Medicine of Sapienza University of Rome according to guidelines established by the Ethical Committee for human subject studies (Helsinki Declaration of 1975, revised in 2008). A detailed description of the samples is reported in Supplementary Table S1.

Among these, about 150 and 52 bodies with documented PMI were considered for the model calibration and validation, respectively, while 22 additional samples contaminated with tissue fragments and documented PMI were processed as unknown samples to evaluate the feasibility of the method. The workflow of the research is summarized in Supplementary Figure S1.

### 2.2. Analytical Procedure

Thirty µL of vitreous humor was analyzed after homogenization by a Perkin Elmer TGA7 thermobalance (Perkin Elmer, MA, USA). Each sample was heated from 20 °C to 800 °C at a heating rate of 10 °C/min as the best resolution rate. The atmosphere was air at a 100 mL/min flow rate. Calibration of the instrument was performed using the Curie-point transition of standard metals, as specified by the equipment recommendations, to ensure accuracy of the method. Each sample was analyzed in triplicate and the resulting average curve was considered for calculations.

### 2.3. Chemometric Analysis

Multivariate statistical analysis was performed by chemometrics, and the thermogravimetric curves were processed by unsupervised techniques such as principal component analysis (PCA) [25,26], while partial least squares discriminant analysis (PLS-DA) [27,28,29] was used to develop the model of prediction for PMI estimation. Optimization of the model was performed by considering the entire dataset of 202 samples and dividing measurements into a training set (about 75% of the samples) and a validation set (about 25% of the samples). In addition, 22 samples contaminated with tissue fragments were processed by the model to evaluate the prediction ability. With respect to previous work, a specific mathematical pretreatment based on column autoscaling of values was applied to reduce or minimize the contribution of those variables (temperatures) in the TG curves (the scores) most affecting the accuracy and the sensitivity of the model.

Diagnostics and acquisition of the thermogravimetric data were carried out by Pyris software (Thermo Fisher Scientific Inc., Waltham, MA, USA), and the ASCII files were processed by Unscrambler X by Camo (Camo Analytics, Oslo, Norway). 

## 3. Results

**Figure 1:**
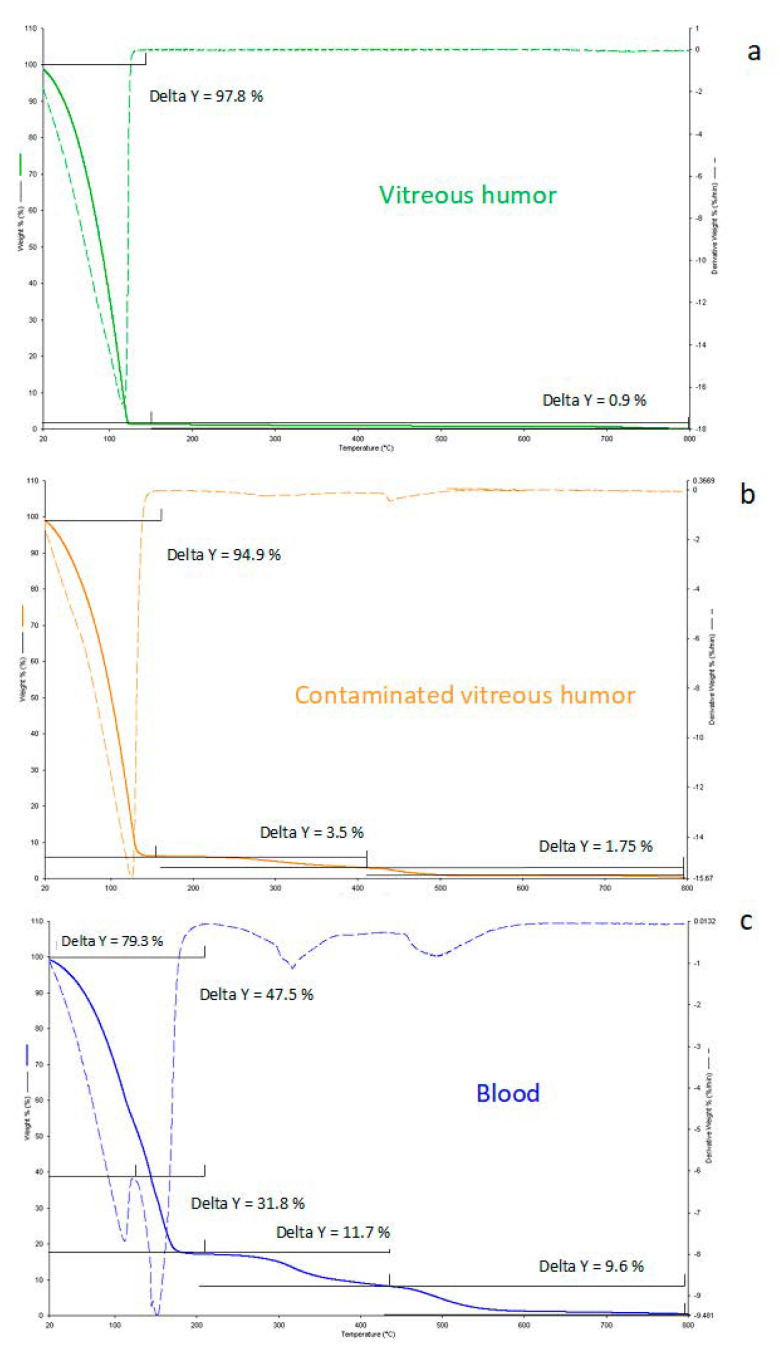
Thermogravimetric (solid lines) and derivative thermogravimetric (DTG) curves (dotted lines) of vitreous humor samples (**a**, green), contaminated vitreous humor samples (**b**, orange), and blood (**c**, blue).

All the collected samples were processed by thermogravimetry, and the characteristic thermogravimetric profile of vitreous humor was compared after heating samples under controlled conditions. As reported in previous work [23,24], no differences in the thermogravimetric curves were observed between the left and right eyes. As a consequence, the mean of the curves from both eyes associated with the same body was considered for chemometric analysis. The investigated samples without contaminations showed a characteristic thermal profile, as reported in Figure 1a, where one main releasing step may be observed, corresponding to the loss of water (about 97.8 ± 0.7%). The thermal decomposition led to a final residue of about 0.9 ± 0.5% (metal oxides at high temperatures). As a consequence of the thermally induced decomposition, a slightly different thermal behavior was observed for those samples contaminated with tissue fragments (Figure 1b), where at least three processes were observed: the first, occurring at 120 °C, corresponded with the release of water, and two more processes were related to decomposition under combustive conditions of the tissue components. No differences in thermogravimetric curves were observed for males and females (*p* values higher than 0.05). Contaminations of vitreous specimens are frequently due to the sampling procedure or to the particular cause of death (eye trauma, hanging, or polytrauma) that provide a perfusion of blood in the vitreous. In Figure 1c, a typical TG curve of blood from a healthy donor (not affected by blood dysfunctions) is reported, showing four main processes: water release in the range of 20–200 °C and decomposition of the corpuscular fraction occurring at 350 °C and 500 °C. In addition, the investigation of the derivative TG curve showed a different water amount distribution of the bulk water (47.5% ± 0.8%) and the bound water (31.8% ± 0.6%) [30,31].

**Figure 2:**
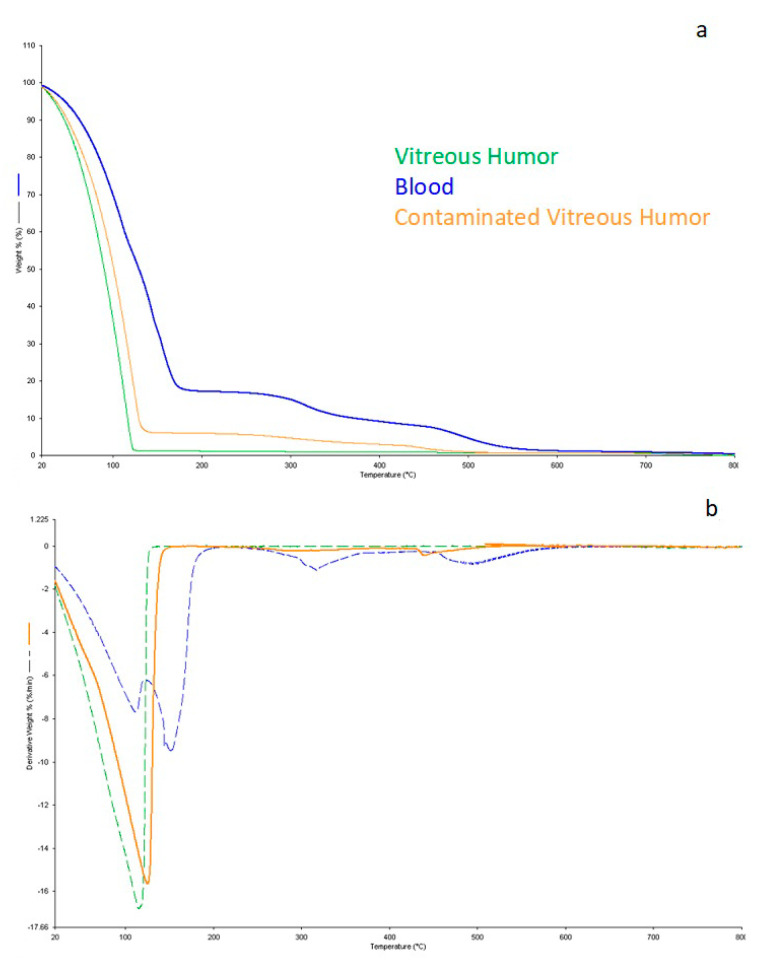
(**a**) Overlapped TG curves of vitreous humor samples (green), contaminated vitreous humor samples (orange), and blood (blue); (**b**) DTG curves of vitreous humor samples (green), contaminated vitreous humor samples (orange), and blood (blue).

By overlapping the three TG curves (Figure 2a), the contamination of vitreous humor with blood was confirmed. In particular, Figure 2b shows that the water contribution in vitreous humor collected after 24 h since death was mainly due to the bulk water [23]. 

In order to provide a method that was as robust as possible and be able to apply it in real forensic situations, a multivariate statistical analysis was performed by chemometric tools only considering the uncontaminated samples and evaluating the prediction ability of the model on the external dataset of contaminated samples.

**Figure 3:**
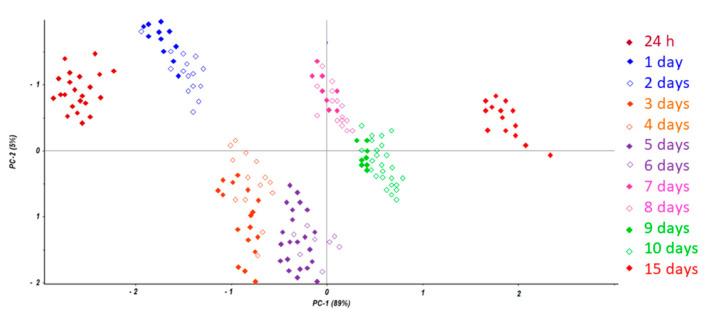
Scores plot from principal component analysis of collected vitreous humor.

With the aim of minimizing the contribution of blood in the instrumental response, data were mathematically pretreated by column autoscaling [32,33] and only temperatures between 20 °C and 250 °C were included in the matrix, as suggested by the analysis of the factor loadings. The resulting scores plot is reported in Figure 3, where colors are used to represent samples belonging to the same group (PMI). The scores plot represents the objects (the samples) in the new vectorial space of principal components and permits visualization of the distribution of samples as a function of the PMI moving along principal component (PC) 1. In fact, samples were found to be distributed in clusters, suggesting the possibility of differentiating samples with the same time since death. This result confirms that the coupling of TGA and chemometrics was able to evaluate the PMI for the estimation of the time since death in forensic pathology.

Based on these results, a prediction model using partial least squares discriminant analysis (PLS-DA) was optimized by dividing the entire dataset into a calibration set (75% of the samples) and an evaluation set (25% of the samples) and considering five latent variables. The model provided for an error of prediction not higher than 0.05% and an overall number of correct classification rate not lower than 70%. The optimized model was used to process the contaminated samples: a detailed description of the samples is reported in Table 1. 

**Figure 4:**
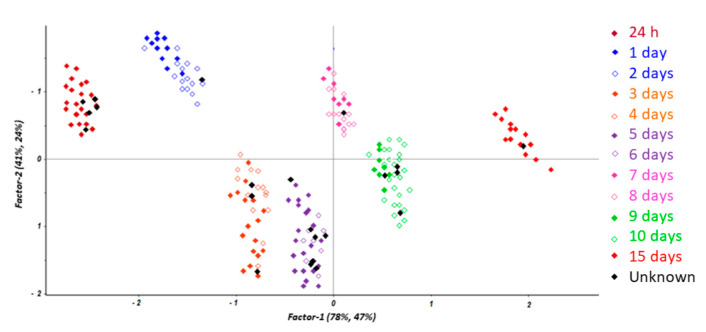
Prediction of the contaminated samples (black) by the partial least squares discriminant analysis (PLS-DA) model.

The resulting scores plot is reported in Figure 4, where a satisfactory prediction was obtained for those samples considered as unknown. In addition, the evaluation of the estimated PMI (22 contaminated samples) versus the calculated values obtained by the model provided a correlation coefficient of about 0.9978, confirming the feasibility of the developed method for correctly estimating the time since death.

## 4. Discussion

To date, the correct determination of the postmortem interval has been somewhat problematic since it has not been supported by a rigorous scientific model. The vitreous humor, thanks to its intrinsic characteristics, is considered the most promising matrix for the optimization of a strict protocol useful in the determination of the postmortem interval.

The literature widely reports different formulas for PMI estimation based on the subjective evaluation of supravital reactions, postmortem lividity, and rigor mortis [3,34,35]. In addition, a number of analytical procedures provide methods for estimating the time since death within a recent time interval (few hours), but officially recognized methods are lacking in the forensic community [36,37,38]. 

The interpretation of the results of this study permits extending the feasibility of the TGA/chemometrics approach, demonstrating that it does not depend on the specific cause of death since contaminated samples may also be processed. In fact, the contribution of the chemometric analysis permitted the construction of an objective predictive model of the postmortem interval, independent of the individual experience of the medical examiner. In particular, the analysis of the vitreous humor by thermoanalytical techniques takes less than two hours, including sampling and without any pretreatment. In this context, the proposed method has a significant impact on costs, due to the reduction of response time and of expenditures related to personnel and consumables. 

With respect to the existing literature, the proposed method of TGA and chemometrics provides the possibility to accurately and rapidly estimate in an objective manner the time since death for up to 15 days. In addition, the results from TGA provide a simultaneous evaluation of the entire chemical composition of the humor sample, not limited to the investigation of the potassium concentration [39,40,41] or the hypoxanthine [42,43,44,45] alone. The possibility of investigating the humor sample without any pretreatment and evaluating the entire chemical composition permit extending the detection of a PMI for up to 15 days.

## 5. Conclusions

In this work, a TGA/chemometric approach was used to estimate the postmortem interval (PMI) in order to provide a comprehensive model able to be used in real forensic cases. In particular, a large dataset of vitreous specimens was collected, and contaminated samples with tissue fragments were processed to evaluate the possibility of extending this approach to the investigation of cadavers after traumatic death or with contaminated vitreous. Results demonstrate that the chemometric analysis permits evaluating the contribution of the contamination and provides for a model able to differentiate vitreous samples according to the PMI in a rapid and accurate way. 

## Figures and Tables

**Table 1:** Detailed description of the contaminated vitreous humor samples.

Sample No.	Age (Years)	Gender	PMI (Days)	Cause of Death
1	29	M	0	Polytrauma
2	56	F	0	Hanging
3	67	M	0	Downfall
4	53	F	0	Car accident
5	47	M	0	Car accident
6	53	M	1	Precipitation shock
7	71	M	3	Precipitation shock
8	35	M	4	Downfall
9	64	M	4	Polytrauma
10	38	F	4	Acute pancreatitis
11	56	M	5	Precipitation shock
12	75	F	5	Downfall
13	54	M	6	Polytrauma
14	36	F	6	Hanging
15	63	F	6	Car accident
16	85	M	6	Car accident - polytrauma
17	75	M	7	Precipitation shock
18	50	F	9	Car accident
19	37	M	10	Car accident
20	65	M	10	Precipitation shock
21	55	F	10	Acute respiratory insufficiency
22	51	F	15	Hanging

## Data Availability

Data available on request due to restrictions eg privacy or ethical reason. The data presented in this study are available on request from the corresponding author.

## Supplementary Materials

The following are available online at https://www.mdpi.com/2075-4418/11/1/121/s1.
