# Supplementary material for: Application of Innovative TGA/Chemometric Approach for Forensic Purposes: The Estimation of the Time since Death in Contaminated Specimens
Source: Diagnostics (Basel). 2021 Jan 14;11(1):121. doi: 10.3390/diagnostics11010121 (PMC7828662; doi:10.3390/diagnostics11010121)
Supplement: Supplementary file 1 [file diagnostics-11-00121-s001.pdf]

Figure S1. Flowchart of the study

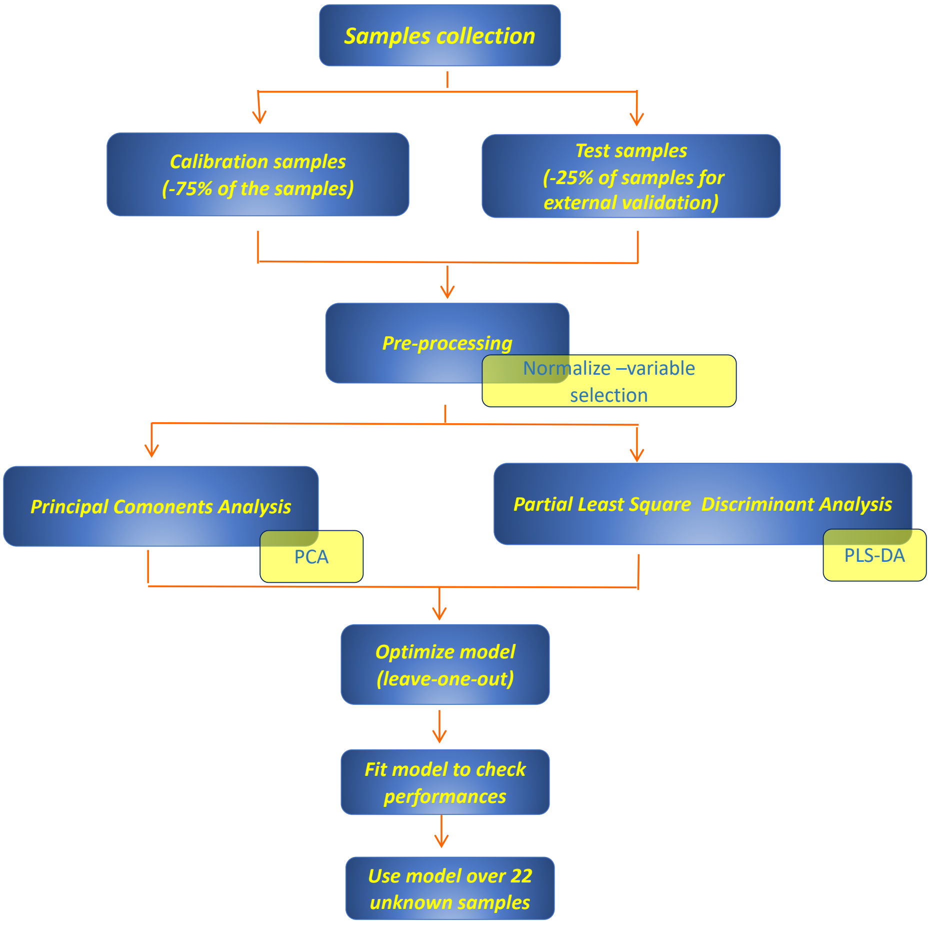

Table S1. Description of the collected vitreous humor samples

Sample n.	Age (years)	Gender	PMI (days)	Cause of death
1	44	F	0	Precipitation shock
2	21	M	0	Drowning
3	45	F	0	Drowning
4	46	F	0	Acute pancreatitis
5	74	M	0	Crushing
6	78	M	0	Hanging
7	69	M	0	Cardiomyopathy
8	82	F	0	Hanging
9	58	M	0	Drowning
10	96	F	0	Hanging
11	32	F	0	Heart attack

12	35	M	0	Car accident
13	47	F	0	Car accident
14	22	M	0	Car accident
15	70	M	0	Cardiomyopathy
16	61	F	0	Pulmonary embolism
17	35	M	0	Pulmonary embolism
18	71	M	0	Pulmonary embolism
19	59	M	0	Precipitation shock
20	38	F	0	Acute respiratory insufficiency
21	57	F	0	Pulmonary embolism
22	62	M	1	Acute cardiorespiratory insufficiency
23	47	F	1	Acute pancreatitis
24	29	M	1	Head trauma
25	55	M	1	Car accident
26	84	M	1	Hanging
27	56	M	1	Precipitation shock
28	93	M	1	Precipitation shock
29	53	F	1	Precipitation shock
30	91	F	1	Hanging
31	77	F	1	Hypothensive shock
32	83	M	1	Hypothensive shock
33	69	M	2	Fire shot
34	50	F	2	Heart attack
35	63	M	2	Pulmonary embolism
36	74	M	2	Acute cardiorespiratory insufficiency
37	41	M	2	Heart attack
38	65	F	2	Head trauma
39	85	M	2	Carbon Monoxide poisoning
40	82	M	2	Car accident
41	76	F	2	Acute cardiorespiratory insufficiency
42	86	F	2	Hanging
43	55	M	3	Heart attack
44	85	F	2	Hanging
45	39	F	2	Car accident
46	52	M	2	Head trauma
47	55	M	3	Vertebral fracture
48	52	F	3	Vertebral fracture
49	64	M	2	Car accident
50	43	F	2	Asphyxia
51	65	M	3	Crushing
52	51	F	3	Head trauma
53	78	M	3	Car accident
54	42	F	3	Head trauma
55	78	M	3	Car accident

56	63	M	3	Car accident
57	75	M	3	Crushing
58	79	F	3	Hanging
59	81	F	3	Hanging
60	88	F	3	Hanging
61	43	F	3	Precipitation shock
62	47	M	3	Precipitation shock
63	32	F	3	Heart attack
64	24	M	3	Heart attack
65	78	M	4	Precipitation shock
66	36	F	4	Heart attack
67	20	M	4	Car accident
68	61	M	4	Acute pancreatitis
69	60	M	4	Car accident
70	82	F	4	Hanging
71	91	F	4	Hanging
72	60	M	4	Acute cardiorespiratory insufficiency
73	84	F	4	Hypothensive shock
74	72	F	4	Pulmonary embolism
75	65	F	4	Acute pancreatitis
76	52	F	4	Pulmonary embolism
77	41	M	4	Heart attack
78	66	F	4	Heart attack
79	78	M	4	Precipitation shock
80	22	M	4	Fire shot
81	27	F	4	Head trauma
82	31	M	4	Car accident
83	87	F	5	Vertebral fracture
84	84	F	5	Vertebral fracture
85	30	M	5	Car accident
86	45	M	5	Butane poisoning
87	58	M	5	Car accident
88	47	M	5	Asphyxia
89	79	F	5	Hypothensive shock
90	82	M	5	Hypothensive shock
91	83	F	5	Hanging
92	83	F	5	Hanging
93	95	F	5	Hanging
94	91	M	5	Hanging
95	44	M	5	Drowning
96	68	F	5	Crushing
97	59	M	5	Crushing
98	62	F	5	Heart attack
99	57	M	5	Car accident

100	63	M	5	Car accident
101	82	F	6	Cardiomyopathy
102	59	F	6	Heart attack
103	56	M	6	Acute cardiorespiratory insufficiency
104	68	F	6	Acute cardiorespiratory insufficiency
105	45	M	6	Acute cardiorespiratory insufficiency
106	64	M	6	Car accident
107	76	M	6	Head trauma
108	59	M	6	Head trauma
109	35	M	7	Fire shot
110	57	F	7	Heart attack
111	46	M	7	Heart attack
112	87	M	7	Vertebral fracture
113	85	M	7	Precipitation shock
114	25	F	7	Acute cardiorespiratory insufficiency
115	65	F	7	Drowning
116	80	M	7	Hypothensive shock
117	81	M	7	Hypothensive shock
118	55	M	7	Hanging
119	54	F	8	Carbon Monoxide poisoning
120	93	F	8	Hanging
121	92	M	8	Hanging
122	67	F	8	Acute pancreatitis
123	70	M	8	Precipitation shock
124	76	M	8	Precipitation shock
125	75	M	8	Hypothensive shock
126	58	F	8	Head trauma
127	75	F	8	Pulmonary embolism
128	31	M	8	Pulmonary embolism
129	70	M	8	Cardiomyopathy
130	97	F	8	Hanging
131	95	M	8	Hanging
132	63	M	8	Crushing
133	85	M	8	Vertebral fracture
134	30	M	9	Car accident
135	75	F	9	Acute cardiorespiratory insufficiency
136	53	F	9	Heart attack
137	42	F	9	Heart attack
138	85	M	9	Precipitation shock
139	62	M	9	Crushing
140	81	F	9	Head trauma
141	50	F	9	Asphyxia
142	49	F	10	Pulmonary embolism
143	51	F	10	Pulmonary embolism

144	42	M	10	Head trauma
145	67	F	10	Head trauma
146	65	M	10	Head trauma
147	64	M	10	Acute pancreatitis
148	32	F	10	Cardiomyopathy
149	51	M	10	Drowning
150	95	M	10	Hanging
151	48	F	10	Car accident
152	63	M	10	Car accident
153	62	M	10	Cardiomyopathy
154	35	F	10	Cardiomyopathy
155	58	F	10	Cardiomyopathy
156	92	F	10	Hanging
157	87	F	10	Vertebral fracture
158	97	M	10	Hanging
159	91	F	10	Hanging
160	94	M	10	Hanging
161	89	F	10	Precipitation shock
162	85	F	10	Precipitation shock
163	51	M	10	Pulmonary embolism
164	75	F	10	Acute pancreatitis
165	70	M	10	Acute cardiorespiratory insufficiency
166	56	M	10	Acute cardiorespiratory insufficiency
167	73	F	10	Acute cardiorespiratory insufficiency
168	81	F	10	Precipitation shock
169	82	M	15	Precipitation shock
170	86	F	15	Hanging
171	74	M	15	Hanging
172	78	M	15	Vertebral fracture
173	76	F	15	Vertebral fracture
174	71	F	15	Vertebral fracture
175	83	M	15	Head trauma
176	62	M	15	Pulmonary embolism
177	75	M	15	Acute pancreatitis
178	68	M	15	Cardiomyopathy
179	63	F	15	Cardiomyopathy
180	45	F	15	Heart attack
181	52	F		Pulmonary embolism
182	63	M		Pulmonary embolism
183	60	F		Heart attack
184	29	M		Heart attack
185	31	M		Drowning
186	74	F		Hanging
187	93	M		Hanging

188	88	F	Hanging
189	96	F	Hanging
190	82	M	Precipitation shock
191	53	M	Cardiomyopathy
192	64	M	Car accident
193	56	F	Car accident
194	87	M	Acute cardiorespiratory insufficiency
195	74	F	Acute cardiorespiratory insufficiency
196	73	M	Acute respiratory insufficiency
197	73	F	Head trauma
198	69	F	Head trauma
199	83	F	Hypothensive shock
200	72	M	Hypothensive shock
201	75	M	Acute pancreatitis
202	63	F	Acute pancreatitis
